# Expectations for Children with Autism Spectrum Disorders or Intellectual Disabilities in Ghana: A Comparison Between Service Providers and Parents

**DOI:** 10.1007/s10803-023-06073-9

**Published:** 2023-08-02

**Authors:** Melissa Washington-Nortey, Adote Anum, Zewelanji Serpell, Yaoying Xu

**Affiliations:** 1 Department of Psychology, King’s College London, Addison House, Guy’s Campus, London, SE1 1UL UK; 2 Department of Psychology, University of Ghana, Accra, Ghana; 3 Department of Psychology, Virginia Commonwealth University, Richmond, USA; 4 Department of Counseling and Special Education, Virginia Commonwealth University, Richmond, USA

**Keywords:** Autism spectrum disorders, Intellectual disabilities, Parents, Service providers, Ghana, Expectations

## Abstract

Little is known about care providers’ expectations for children with autism spectrum disorders (ASD) and intellectual disabilities (ID) in Ghana. This study used group concept mapping (n = 9) and a quantitative survey (n = 128) to explore and compare service providers’ and parents’ expectations for children with ASD or ID. Data were analyzed using hierarchical clustering procedures and Multivariate Analysis of Variance (MANOVA). Concept mapping results revealed several expectation clusters, including *independence, love and acceptance, equal social rights and opportunities*, and *professional and caregiver training*. MANOVA results revealed significant differences between parents, teachers, and healthcare providers in their perceptions of the importance and likelihood of a child achieving these expectations. Results are discussed in reference to the cultural context, and implications are outlined.

Approximately 52.9 million children under five years of age have a developmental disability, and 95% of these children live in Low and Middle-Income Countries (LMICs) (Olusanya et al., 2018). Developmental disabilities are childhood onset disabilities like cerebral palsy, attention deficit hyperactivity disorder (ADHD), intellectual disabilities, autism spectrum disorders, and other learning disorders that cause impairments in physical, learning, or behavioral functioning (CDC, 2022). Autism spectrum disorders (ASD) and intellectual disabilities are among the most common developmental disabilities and have received much attention in research studies, including studies conducted in LMICs. However, in Ghana, a lower-middle-income country on the West African coast, much remains unknown about the care of children with ASD or ID (Wireko-Gyebi & Ashiagbor, 2018).

Service providers are critical in caring for children with developmental disorders like ASD and ID, and education and healthcare represent two vital service provision sectors. Research targeting teachers in Ghana has focused mainly on policy and practice-related topics (Butakor et al., 2020; Monico et al., 2020). Work with healthcare providers has mainly focused on parents’ experiences navigating healthcare systems and the amount, type, and quality of services available (Abrokwah, 2018; Lamptey, 2020). However, much less attention has been paid to examining the perceptions of healthcare providers working with children with developmental disabilities^1^ and their families. And except for a single study (Washington-Nortey & Serpell, 2021), Ghanaian parents’ expectations for their children with ASD or ID have not been examined. Service providers’ perceptions, attitudes, and expectations may influence service delivery to children with developmental disabilities and their families (Iezzoni et al., 2021) and affect children’s developmental trajectories. Further, differences in parent and service providers’ perspectives may increase parental stress, influence parental involvement in service provision and care management, impact the development of collaborative relationships, and affect the family quality of life (Đorđević et al., 2021; Liptak et al., 2006). The current study investigates service providers’ expectations for children with ASD or ID in Ghana and compares their perspectives with that of parents.

To better reflect the Ghanaian context and our sample, we combined perspectives on ASD and ID. First, in Ghana, due to resource and awareness limitations, most children identified and diagnosed with ASD or ID are also likely to exhibit significant impairments in language, motor, or cognitive domains. Few cases of highly verbal and non-cognitively impaired children come to the attention of teachers and healthcare workers, and very few professionals are trained to diagnose nuanced presentations of these conditions (Cardon, 2017). Second, children with more severe symptoms, in Ghana, are more likely to receive educational services in segregated settings like special schools (Botts & Owusu, 2013). These include several recently established autism centers that have helped to increase general awareness of ASD in the country and comprised our principal sampling frame. Third, studies show that children with more severe ASD or ID exhibit similar symptoms that can make distinguishing between these conditions challenging (Pedersen et al., 2017; Thurm et al., 2019). Therefore, we expected perceptions to be shaped by personal experience and the current service provision strategy, which might increase awareness about more severe symptoms as they are more likely to be identified.

## Service Providers and Children with ASD or ID

### Service Providers in Schools

In Ghana, most studies about service providers for children with developmental disabilities like ASD and ID in schools investigate topics related to inclusive education. For instance, several studies have been conducted on teachers’ attitudes and beliefs about inclusive education using varied instruments (Butakor et al., 2020; Monico et al., 2020). Whereas attitude-focused studies probe issues related to teacher’s willingness to engage in the practice (e.g., whether teachers are willing to revise the curriculum to meet the needs of all students in their classrooms irrespective of their disability status), belief-focused studies assess teachers’ views on the practice (e.g., whether teachers believe that inclusive education benefits all students). In some cases, there are overlaps in the study definitions of these constructs (e.g., Vanderpuye et al., 2020).The evidence on teachers’ attitudes towards and beliefs about inclusive education is mixed.

Studies engaging exclusively Ghanaian participants show that teacher attitudes are reasonably positive and may be similar across genders regarding inclusive education (Butakor et al., 2020). For example, Ghanaian teachers are willing to adapt their curricula and communication techniques (Butakor et al., 2020), and engage in team teaching to meet their students’ diverse needs (Vanderpuye et al., 2020). However, comparison studies with teachers from other countries like Spain and Germany indicate that Ghanaian teachers may have less positive attitudes towards the practice (Monico et al., 2020).

Studies on beliefs show that Ghanaian teachers may be less optimistic about the value of inclusive education compared to teachers in Spain and Germany. For example, fewer Ghanaian teachers deemed it appropriate for children with severe ID to spend all or most of their time in a general education classroom (Monico et al., 2020). Preliminary evidence also indicates that male teachers in Ghana may hold more negative beliefs than female teachers even though positive beliefs are similar across genders (Butakor et al., 2020). For instance, Butakor and colleagues (2020) found no significant differences between male and female teachers on the (positive) belief that children with disabilities can learn in general education environments when curricula are adapted to their needs. However, male teachers were more likely to believe that segregated education is more appropriate for these children.

Beliefs about the impact of inclusive education on children with developmental disabilities like ASD and ID may be correlated with teachers’ attitudes (Vanderpuye et al., 2020). Moreover, a teacher’s perception of these children’s capacity for learning and/or their expectations for these children may affect their attitudes and willingness to engage in inclusive practices and other strategies. However, no Ghanaian studies have examined teachers’ expectations for children with ASD or ID.

### Service Providers in Healthcare Centers

Most Ghanaian-based studies about healthcare providers of children with developmental disabilities like ASD and ID have investigated the amount, type, and quality of service. Specialized knowledge among healthcare providers about developmental disabilities is low (Dassah et al., 2019). Wireko-Gyebi and Ashiagbor (2018) found that among pediatric and psychiatric nurses in Kumasi, one of the largest metropolitan areas in Ghana, many nurses only learned about ASD through patient encounters. There are a limited number of skilled healthcare providers in Ghana, and paraprofessionals often assist in caring for children with developmental disabilities like ASD and ID. However, paraprofessionals (i.e., individuals with some training, but lacking full qualifications for a position), have limited knowledge, receive little in-service training, and, not surprisingly, report challenges associated with diagnosing, medicating, and providing general care to these children and their families (Odongo et al., 2021). Several studies also document that parents face challenges accessing care for their children with developmental disabilities (Abrokwah, 2018; Badu et al., 2016; Lamptey, 2020).

Beyond documenting the amount, type, and quality of care available to children with developmental disabilities like ASD and ID in Ghana, a few studies have examined perceptions held by healthcare providers about their patients. For instance, Acheampong et al. (2021) qualitatively examined the perceptions of healthcare providers working with patients with disabilities. They noted that the healthcare providers perceived these individuals as selfish and inconsiderate, lacking self-esteem, stressed, challenging to communicate with, and aggressive and predisposed to violent outbursts. Although participants in the study referenced mostly adults with disabilities, the extant literature on beliefs related to developmental disabilities and the general population’s attitudes, including healthcare providers (Baffoe, 2013), suggests that similar perceptions may prevail when interacting with children with developmental disabilities.

Historically, developmental disabilities such as ASD and ID were perceived as consequences of sinful acts committed by parents (Kassah et al., 2012). Therefore, children born with developmental disabilities and their families were isolated from society, heavily stigmatised and discriminated against (Kassah et al., 2012). Although these etiological beliefs are gradually being replaced by scientific evidence and explanations among educated individuals, the negative attitudes and behaviors associated with developmental disabilities persist (Oti-Boadi et al., 2020). Healthcare providers’ expectations for these children are also critical as expectations have been linked to actions and consequent outcomes in previous studies (Simpkins et al., 2012). Unfortunately, no studies have examined healthcare providers’ expectations for children with ASD or ID in Ghana. However, evidence from other African countries indicates that expectations for children with ASD or ID, were consistently low even after engaging in interventions meant to increase knowledge of different disability categories (Tilahun et al., 2019). This finding contrasts with findings from Ghana, indicating that parents of children with ASD or ID have high expectations for their children across multiple domains (Washington-Nortey & Serpell, 2021).

## The Current Study

Given the potential implications of low expectations on service provision for children with developmental disabilities and their trajectories, it is critical to investigate service provider expectations for children with developmental disabilities like ASD and ID. Additionally, it is essential to assess how these service provider expectations compare with parent expectations as divergent goals can hinder the implementation of strategies to improve the trajectories of children with developmental disabilities. Therefore, this study specifically examined teachers’ and healthcare providers’ expectations for children with ASD or ID. It also compared these service providers’ expectations with parents’ expectations on two different criteria. The specific research questions were:

1. What are Ghanaian service providers’ expectations for children with ASD or ID?
2. Do service providers and parents of children with ASD or ID differ in their perceptions of the importance of specific expectation domains?
3. Do service providers and parents of children with ASD or ID differ in their perceptions of the likelihood that children with ASD or ID will attain each of the expectation domains identified?

## Method

### Study Design

We used a mixed-methods exploratory sequential design (Creswell & Plano Clark, 2011) to assess service providers’ and parents’ expectations for children with ASD or ID. It comprised concept mapping (Kane & Trochim, 2007)—a theory-based, collaborative approach that gathers key stakeholder perspectives on topics in a focus group setting—followed by a quantitative survey based on data from the concept mapping process.

### Participants and Sampling

Nine service providers, including general and special education teachers (*n* = 4) and teaching aids (*n* = 2), healthcare providers (*n* = 1), and other community stakeholders (*n* = 2), participated in the concept mapping focus group meeting. All were Ghanaian and Christian, and most (66%) had attained a bachelor’s degree or higher. The mean age was 39.22 years (*SD* = 11.60). On the other hand, 128 service providers and parents completed the follow-up survey developed using data from the concept mapping process. They included special education teachers (*n* = 17), teaching aids (*n* = 22), healthcare providers (*n* = 38), and parents of children with ID or ASD, as confirmed by institutional authorities (n = 51). Teachers served children under 18 years and health workers served a broader age range of children, as specialist providers are scarce in the country. Details of their demographic characteristics are presented in Table 1. This strategy allowed us to investigate service providers’ expectations and examine parents’ views of these expectations to assess similarities and differences. In a separate paper, we exclusively explore parents’ expectations (Washington-Nortey & Serpell, 2021). We did not combine parents and service providers in the same concept mapping group. We anticipated that parents would have a more subjective view of expectations, whereas service providers were more likely to hold objective views. Therefore, we kept these groups separate, to ensure each group felt comfortable sharing these perspectives.

**Table 1:** Demographic Characteristics of Follow Up Survey Respondents

		Special Ed Teachers		Special Ed Teaching Aids		Healthcare providers		Parents	
		N (17)	%	N (22)	%	N (38)	%	N (51)	%
Sex	Male	5	29%	4	18%	14	37%	21	41%
	Female	16	65%	18	82%	24	63%	24	47%
	Missing	1	6%	--	--	--	--	6	12%
Ethnicity									
	Akan	5	29%	6	27%	19	50%	23	45%
	Ga-Dangme	0	--	3	14	4	11%	7	14%
	Other	12	71%	13	59%	15	39%	20	39%
	Missing	0	--	0	--	0	--	1	2%
Religious Affiliation									
	Christian	14	82%	18	81%	32	84%	43	84%
	Other	1	6%	2	10%	6	16%	8	16%
	Missing	2	12%	2	9%	0	–	0	–
Highest Level of Education									
	None-Junior high school	0	--	1	5%	0	--	6	12%
	Senior high school/equivalent	0	--	10	45%	1	3%	7	14%
	Professional training/diploma	0	--	5	23%	18	47%	11	22%
	Bachelor’s Degree and above	17	100%	6	27%	19	50%	25	48%
	Missing	0	--	0	--	0	--	2	4%
Mean age (Standard Deviation)		36.79 (9.67)		27.35 (8.71)		28.69 (4.96)		41.91 (8.03)	

We used purposive, and snowball sampling methods to recruit these participants from educational institutions, local hospitals, and other organizations serving children with developmental disabilities and their families. Whereas purposive and convenience sampling strategies helped attain eligible participants, snowball methods helped us increase the sample size and access participants who would otherwise be difficult to reach.

### Procedure

Concept mapping steps included (1) preparation of a focus group prompt and demographic questionnaire; (2) identification and selection of participants; (3) generation of ideas or statements; (4) sorting and rating of idea statements; (5) analyses and visual representation of the data; and (6) interpretation (Kane & Trochim, 2007). The researchers completed steps 1,2, 5 and 6, whereas steps 3 and 4 were completed by participants during the concept mapping session. First, we used a tailored prompt developed in consultation with graduate students, field experts, and parents of children with developmental disabilities in this study. It stated, “one dream/hope or expectation that you have for a child with autism or intellectual disability is___”?

**Fig. 1:**
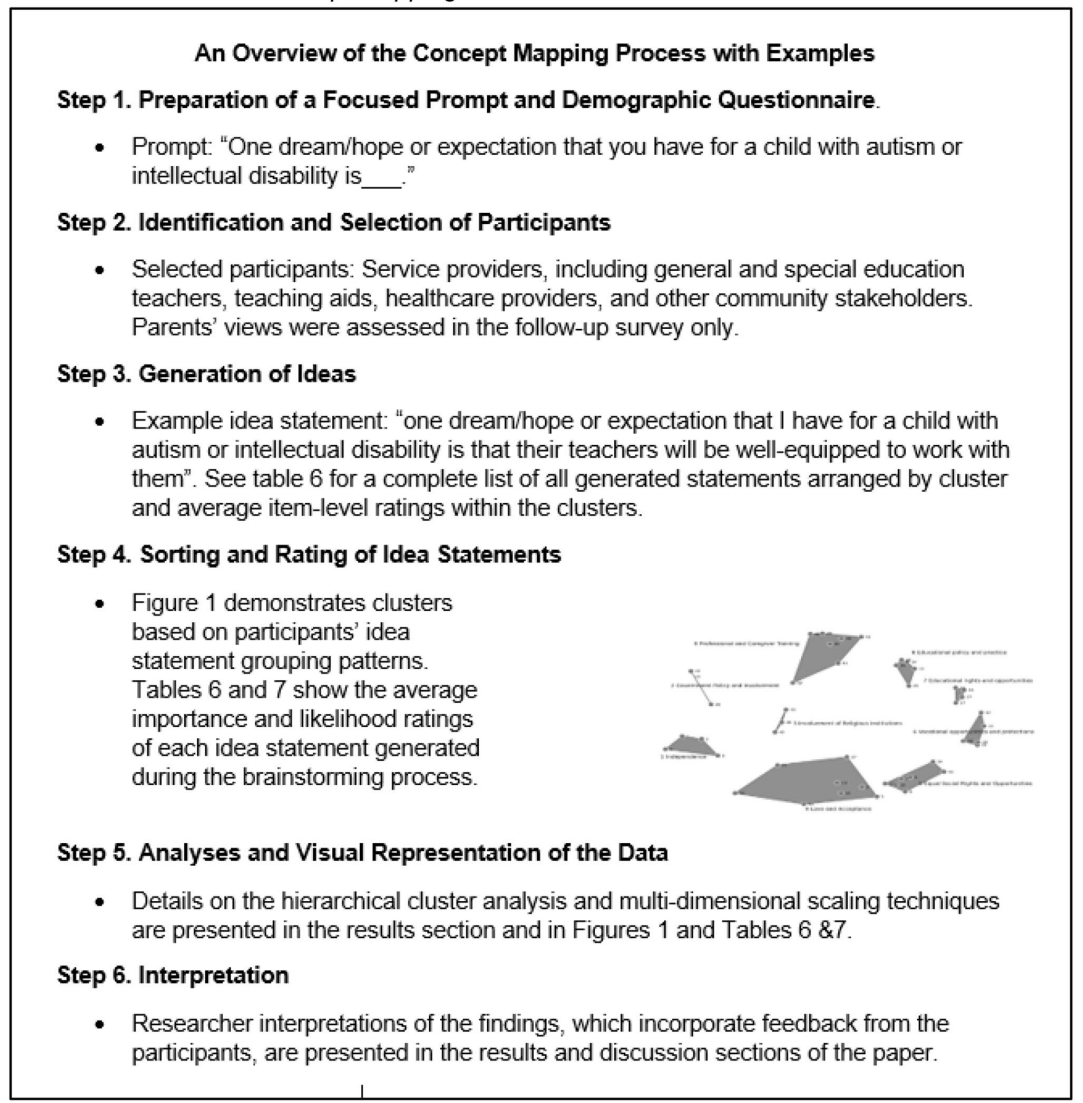
Research Box on the Concept Mapping Process

In step 2, we identified service providers likely to engage with children with ASD or ID in their line of work. These included general and special education teachers, special education teaching aids, healthcare providers, and other professionals likely to work or regularly interact with our sample. Steps 3 and 4 took place in a focus group meeting held at a central location in Accra, the capital city. There, service providers engaged in a brainstorming process using the specified prompt. Each participant shared as many unique expectations (i.e., one previously unmentioned expectation) as desired during the session. The session was organized in English as it is the official Ghanaian language, and all participants were proficient in the language. As such, there was no need to translate statements during or after the session. At the end of the brainstorming process, there were 46 unique expectation statements. They included statements like, “one dream/hope or expectation that I have for a child with autism or intellectual disability is *that they will be loved by society*” and “one dream/hope or expectation that I have for a child with autism or intellectual disability is *that their teachers will be well-equipped to work with them*” (see Tables 2 and 3 for a more comprehensive list of each statement generated during the session). After the brainstorming process, each service provider received a pile of cards equal to the number of expectation statements generated in the session (i.e., each card listed one expectation statement shared during the brainstorming process). Two service providers missed the brainstorming component of the meeting. However, they agreed with all the previously generated statements and participated in the sorting and rating process. Each person divided (sorted) their 46 cards into different groups based on themes they felt were captured by different sets of cards. However, they could not create a group containing a single card or a group containing all 46 cards. Therefore, each card group could contain at least two cards and at most 45 cards. Next, participants received all 46 statements in a 5-point Likert survey format and rated each statement on two criteria: (1) the perceived importance and (2) the perceived likelihood of attainment for a child with ASD or ID. Higher ratings indicated that the participants perceived a specific statement as important or likely. The focus group meeting lasted approximately three hours, and service providers received the local currency equivalent of $10.00 as compensation. Figure 1 illustrates the concept mapping process with examples.

**Table 2:** Average Importance Ratings for Cluster Compositions of the Ghana Service Provider Focus Group

Cluster Labels and Contents	Avg. Importance Ratings
**Independence**	**4.63**
1.To be independent.	4.78
2. To know what they need and have that need be respected once it is in their best interest.	4.78
3. To be able to communicate their wishes to others.	4.63
4. For them to come to personal faith in the Lord Jesus Christ.	4.33
**Government policy and involvement**	**4.52**
23. That the government will allocate more funds for children with special needs.	4.78
36. They will be motivated to achieve anything they desire.	4.44
22. There will be government policies to cover the hospital bills of children with special needs.	4.33
**Involvement of religious institutions**	**4.52**
44. Religious institutions will be involved in raising awareness about the needs of these children.	4.56
45. Religious institutions will welcome children with special needs.	4.56
46. Religious institutions will give them opportunities to express themselves publicly.	4.44
**Vocational opportunities and protections**	**4.44**
31. They will not be discriminated against in the workplace.	4.67
29. That they will be treated fairly in the workplace.	4.44
30.That they will be paid fairly in the workplace.	4.44
12. They should be given opportunities to develop vocational skills.	4.33
13. They should be given opportunities to work.	4.33
**Educational policy and practice**	**4.42**
14. That their teachers will be well-equipped to work with them.	4.89
15.That their teachers will desire to bring out the best in them.	4.44
28. Every mainstream school will have at least one teacher trained to work with children with special needs.	4.44
26. That teachers in mainstream schools will be trained to work with children with special needs.	4.22
25. They will be accepted or included in mainstream schools.	4.11
**Professional and caregiver training**	**4.41**
38. Their caregivers will be educated about their dietary needs/restrictions based on their specific disabilities and health needs.	4.67
39. That their parents and other relatives will be trained to effectively handle them.	4.67
35. Health professionals will be trained specifically to work with children with special needs.	4.44
41. The general society will be educated on how to interact with children with special needs.	4.44
32. They will be given preferential treatment where necessary.	4.22
40. That their parents will receive periodic supervision in the care of their children with special needs.	4.22
42. That security personnel will be trained on how to interact or handle persons with special needs.	4.22
**Equal Social Rights and Opportunities**	**4.40**
6. That they will not be discriminated against by members of society.	4.56
8. For society to see them as humans (not second-class citizens).	4.56
43. That children/persons with special needs will not be abused by security personnel.	4.56
7. That they would be welcome in all social settings.	4.44
11. They should be given the same meals as other members of the society who are seen as “normal”.	4.33
10. For them to have the same opportunities as everyone else in society.	4.22
24. They will have access to all social amenities.	4.11
**Educational rights and opportunities**	**4.39**
16. They will have access to an appropriate environment for learning.	4.44
17. They will have access to appropriate equipment, facilities and resources.	4.44
27.That school classrooms and other facilities will be designed to accommodate children with special needs.	4.44
34. They will be allowed to attain the highest level of education possible.	4.22
**Love and Acceptance**	**4.29**
19. That they will be loved by parents and relatives.	4.89
20. That they will be taught to love themselves as they are.	4.56
18. That they will be loved by society.	4.44
5. To be accepted as full members of society.	4.33
9. For them to have the same rights as everyone else.	4.33
33.They will be treated with empathy not sympathy.	4.22
37. All their nutritional needs will be met (i.e., they will have enough food).	4.22
21. They will be allowed to start families of their own.	3.33

*N.B. Statement in bold represent cluster labels and all numbers are points on the respective maps

**Table 3:** Average Likelihood Ratings for Cluster Compositions of the Ghana Service Provider Focus Group

Cluster Labels and Contents	Avg. Likelihood Ratings
**Involvement of religious institutions**	**4.15**
45. Religious institutions will welcome children with special needs.	4.44
44. Religious institutions will be involved in raising awareness about the needs of these children.	4.22
46. Religious institutions will give them opportunities to express themselves publicly.	3.78
**Independence**	**4.14**
1.To be independent.	4.22
3. To be able to communicate their wishes to others.	4.22
2. To know what they need and have that need be respected once it is in their best interest.	4.11
4. For them to come to personal faith in the Lord Jesus Christ.	4.00
**Educational policy and practice**	**3.96**
14. That their teachers will be well-equipped to work with them.	4.44
15.That their teachers will desire to bring out the best in them.	4.00
26. That teachers in mainstream schools will be trained to work with children with special needs.	3.89
28. Every mainstream school will have at least one teacher trained to work with children with special needs.	3.78
25. They will be accepted or included in mainstream schools.	3.65
**Professional and caregiver training**	**3.84**
39. That their parents and other relatives will be trained to effectively handle them.	4.44
35. Health professionals will be trained specifically to work with children with special needs.	4.33
38. Their caregivers will be educated about their dietary needs/restrictions based on their specific disabilities and health needs.	4.11
41. The general society will be educated on how to interact with children with special needs.	3.67
32. They will be given preferential treatment where necessary.	3.56
40. That their parents will receive periodic supervision in the care of their children with special needs.	3.44
42. That security personnel will be trained on how to interact or handle persons with special needs.	3.33
**Love and Acceptance**	**3.83**
19. That they will be loved by parents and relatives.	4.44
20. That they will be taught to love themselves as they are.	4.22
9. For them to have the same rights as everyone else.	3.89
5. To be accepted as full members of society.	3.67
37. All their nutritional needs will be met (i.e., they will have enough food).	3.89
18. That they will be loved by society.	3.67
33.They will be treated with empathy not sympathy.	3.56
21. They will be allowed to start families of their own.	3.33
**Educational rights and opportunities**	**3.69**
16. They will have access to an appropriate environment for learning.	4.00
17. They will have access to appropriate equipment, facilities and resources.	3.89
27.That school classrooms and other facilities will be designed to accommodate children with special needs.	3.44
34. They will be allowed to attain the highest level of education possible.	3.44
**Equal Social Rights and Opportunities**	**3.68**
43. That children/persons with special needs will not be abused by security personnel.	4.00
8. For society to see them as humans (not second-class citizens).	3.89
6. That they will not be discriminated against by members of society.	3.67
11. They should be given the same meals as other members of the society who are seen as “normal”.	3.67
24. They will have access to all social amenities.	3.67
7. That they would be welcome in all social settings.	3.44
10. For them to have the same opportunities as everyone else in society.	3.44
**Vocational opportunities and protections**	**3.77**
12. They should be given opportunities to develop vocational skills.	4.00
30.That they will be paid fairly in the workplace.	3.89
13. They should be given opportunities to work.	3.78
31. They will not be discriminated against in the workplace.	3.67
29. That they will be treated fairly in the workplace.	3.50
**Government policy and involvement**	**3.70**
22. There will be government policies to cover the hospital bills of children with special needs.	3.89
36. They will be motivated to achieve anything they desire.	3.78
23. That the government will allocate more funds for children with special needs.	3.44

*N.B. Statement in bold represent cluster labels, and all numbers are points on the respective maps

Phase 2—the follow-up survey—began after the focus group meeting. It involved making additional copies of the Likert surveys based on the 46 unique statements and distributing these surveys more widely to service providers and parents of children with ASD and ID. Similar to focus group participants, the follow-up survey respondents rated each unique expectation statement on the importance and likelihood criteria.

The project was led by the first author—an advanced graduate student with substantive training and experience with qualitative and quantitative research methods—born and raised in Ghana and, therefore familiar with local customs and proficient in its dominant local languages. The student was closely supervised by two senior researchers of African descent: a Ghanaian-origin member of faculty and researcher from the University of Ghana, and a Zambian-origin developmental scientist with substantial experience using concept mapping methods. The project-lead also self-trained in the concept mapping process with the concept-mapping method developers and participatef in an hour webinar they hosted. During data collection and data analysis, the project lead accessed expert consultation provided by the developers on an as-needed basis. Ethics approval for this project was awarded by the University institutional review board of the first author (HM20016759_Ame1).

### Data Analytic Procedure

We used the group concept mapping software (The Concept System®, 2020) to analyze concept mapping data. Concept systems use hierarchical cluster analyses and multidimensional scaling techniques to generate multiple cluster solutions based on participants’ sorting patterns. Statements more likely to be grouped by different participants are nearer each other on the cluster map and vice versa. The process yields a stress value—a fit index—demonstrating the generated maps’ fit with the original similarity matrix of the data. Stress values range between 0.1 and 0.35, with lower values indicating better fit. The generated cluster maps also incorporate data from the rating process. Thus, each cluster on the map also has information on the average importance and likelihood rating for each statement in the cluster and the average importance and likelihood rating of the entire cluster. We used several criteria to select an appropriate cluster solution: (1) the solution’s fit with the data, (2) the likelihood that different participants grouped statements in the clusters as indicated by the results of a spanning analysis provided by the software, (3) the group labels participants suggested during sorting, and (4) the conceptual clarity of each cluster’s statements. We carefully examined component statements in each cluster to create cluster-specific definitions that captured the essence of each cluster fully. The results from the concept mapping process informed our analysis of the follow-up survey data.

Data from the follow-up survey were analyzed using the Statistical Package for the Social Sciences (SPSS) version 26. Survey data were aggregated based on the clusters that emerged from the concept mapping process. Then, we used Multivariate Analysis of Variance strategies to examine differences between parents and service providers on their perceptions of the importance of each expectation cluster and the likelihood of attainment by a child with ASD or ID. In the analyses, type of care provider (parent, teacher, or healthcare provider) served as the independent variable, and ratings on the importance and likelihood of each aggregate cluster served as the dependent variables. After analysing the data, preliminary results were presented to the participants to check whether the results aligned with their perspectives and had been accurately interpreted. Participants confirmed our results aligned with their perspectives.

## Results

### Concept Mapping Results and Description of Expectation Clusters

**Fig. 2:**
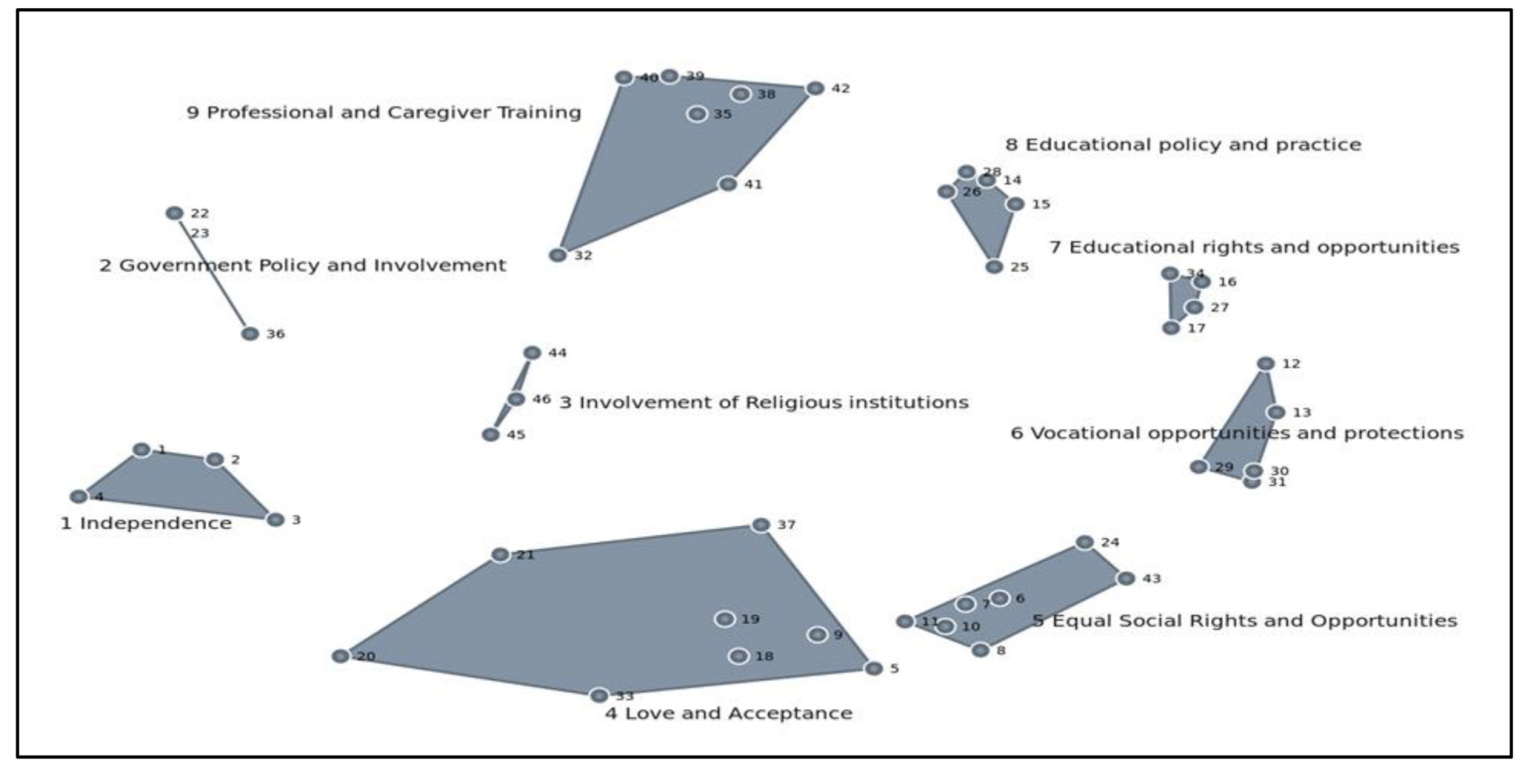
Cluster Map for Ghanaian Service Providers

Multi-dimensional scaling and cluster analysis produced a series of cluster maps after nine iterations with a stress value of 0.2903. We identified a nine-cluster solution as the best fit for the data based on the criteria outlined above. As seen in Fig. 2, the clusters were labeled: *independence, love and acceptance, equal social rights and opportunities*,

*vocational rights and protections, educational rights and opportunities, educational policy and practice, government policy and involvement, involvement of religious institutions, and*

*professional and caregiver training*.

The *independence* cluster included statements about choice and autonomy. The *love and acceptance* cluster comprised statements on self-love and love from family members and society. The *equal social rights and opportunities* cluster contained statements about equitable treatment and access to social gatherings and amenities. The *educational policy and practice* cluster included statements about teacher training and strategies to facilitate inclusive education, and the *educational rights and opportunities* cluster contained statements about the educational environment to which children should be exposed. The *vocational opportunities and protections* cluster described workplace opportunities like access to jobs and fair wages that service providers desired children with ASD or ID receive, and the *government policy and involvement* cluster contained statements about expectations that required government intervention. The last two clusters, *involvement of religious institutions* and *professional and caregiver training*, highlighted ways religious institutions could facilitate inclusion and the training needs of their caregivers and professionals responsible for their care.

### Average Importance and Likelihood Ratings of the Expectation Clusters

On the importance criteria, the *independence* cluster had the highest importance rating, whereas the *love and acceptance* cluster had the lowest rating. However, on the likelihood criteria, service providers rated *religious involvement* more highly than all other clusters and accorded *equal social rights and opportunities* the lowest rating. The average importance and likelihood ratings for the nine clusters are shown in Table 4. However, we provide two more detailed statement-level tables for the average importance and likelihood ratings of each cluster’s items in Tables 2 and 3, respectively at the end of the manuscript.

**Table 4:** Cluster Compositions for Ghana Service Provider Focus Group

Cluster Labels and Contents	Avg. Importance Ratings	Avg. Likelihood Ratings
Independence	4.63	4.14
Love and Acceptance	4.29	3.83
Equal Social Rights and Opportunities	4.40	3.68
Educational policy and practice	4.42	3.96
Educational rights and opportunities	4.39	3.69
Vocational opportunities and protections	4.44	3.77
Government policy and involvement	4.52	3.70
Involvement of religious institutions	4.52	4.15
Professional and caregiver training	4.41	3.84

*N.B. Statement in bold represent cluster labels and all numbers are points on the respective maps

### The Follow-up Survey: Comparisons Between Parents and Service Providers

Before comparing service providers’ ratings to parent ratings on any of the nine expectation clusters, it was important to ascertain whether differences existed between the service provider groups. A MANOVA test showed significant differences in service provider ratings on the importance criteria (Wilks’ ˄=0.418, *F*_(18,62)_ = 1.89 *p* = .034, partial ɳ^2^=0.354). Healthcare providers on each aggregate cluster evidenced lower mean ratings than special education teachers and special education teaching aids. Further examinations with post hoc analysis revealed a significant difference between healthcare providers and special education teachers in one cluster: *government policy and involvement*. There were no significant differences between teachers and teaching aids on any aggregate clusters. A second MANOVA test, used to probe differences between service providers on the likelihood ratings of each aggregate cluster, was not significant (Wilks’ ˄=0.624, *F*_(18,76)_ = 1.12, *p* = n.s, partial ɳ^2^=0.210). However, since healthcare providers differed from teachers on at least one cluster on the importance criterion, we could not treat service providers as a single group. Instead, we separated service providers into two groups—healthcare providers and teachers—for the main analysis.

The main analysis comprised two separate MANOVAs. The MANOVA assessing differences between parents, teachers, and healthcare providers on the importance criteria of each aggregate cluster was significant (Wilks’˄=0.528, *F*_(18,124)_ = 2.60, *p* = .001, partial ɳ ^2^=0.274) as was the MANOVA assessing differences on the likelihood criteria (Wilks’ ˄=. 608, F _(18,150)_ = 2.351, p = .003, partial ɳ ^2^=0.220). The means and standard deviations for parents, teachers, and healthcare providers on the importance and likelihood criteria of each aggregate cluster are presented in Table 5 and summaries from the between-subjects tests are presented in Tables 6 and 7.

**Table 5:** Means and Standard Deviations for Importance and Likelihood Aggregate Clusters in the follow-up survey

Aggregate Cluster Name			Importance		Likelihood	
			*M*	SD	*M*	SD
Independence						
	Parents		18.90	1.680	18.05	2.877
	Sp. Ed teachers		17.74	1.910	15.38	3.843
	Healthcare providers		16.91	1.411	16.28	2.75
	Total		17.97	1.856	16.79	3.31
Government policies						
	Parents		13.84	1.440	12.57	2.48
	SpEd teachers		14.37	1.012	11.63	2.89
	Healthcare providers		13.35	1.43	11.08	2.87
	Total		13.82	1.38	11.87	2.76
Religious involvement						
	Parents		13.90	2.34	13.00	2.686
	SpEd teachers		13.74	1.79	11.71	3.72
	Healthcare providers		13.43	1.65	12.24	2.63
	Total		13.71	1.99	12.42	3.00
Love and acceptance						
	Parents		37.84	2.22	36.84	3.77
	SpEd teachers		36.84	2.14	32.04	6.40
	Healthcare providers		36.17	2.87	31.72	5.22
	Total		37.05	2.50	34.01	5.56
Equal social rights and opportunities						
	Parents		33.58	2.13	31.68	4.53
	SpEd teachers		33.32	2.36	26.67	6.39
	Healthcare providers		31.87	3.43	26.56	5.601
	Total		32.97	2.73	28.79	5.91
Vocational opportunities						
	Parents		23.90	2.02	22.73	3.21
	SpEd teachers		24.63	0.60	19.33	4.90
	Healthcare providers		23.00	2.32	18.88	3.82
			Importance		Likelihood	
			*M*	*SD*	*M*	*SD*
	Total		23.80	2.00	20.66	4.28
Educational opportunities						
	Parents		18.94	1.44	18.27	2.26
	SpEd teachers		19.00	1.29	15.33	4.11
	Healthcare providers		17.91	1.70	15.64	3.70
	Total		18.63	1.55	16.69	3.54
Educational policy						
	Parents		23.94	1.90	23.22	2.86
	SpEd teachers		23.84	1.57	19.79	4.46
	Healthcare providers		22.65	2.40	19.60	3.81
	Total		23.51	2.06	21.21	4.00
Parent and professional training						
	Parents		32.96	2.84	30.97	4.58
	SpEd teachers		33.42	1.84	28.38	5.79
	Healthcare providers		31.78	2.84	28.20	4.67
	Total		32.70	2.67	29.44	5.09

**Table 6:** MANOVA Examining Differences in Ratings on Importance Criterion by Participant Group

Criterion	Predictor	Aggregate Cluster Name	*df*		*F*	Partial ɳ^2^
Importance						
	Part. Type	Independence		2	9.68**	0.217
		Government policy		2	3.02	0.079
		Religious involvement		2	0.36	0.010
		Love and acceptance		2	3.21	0.084
		Equal social rights and opportunities		2	2.95	0.078
		Vocational opportunities		2	3.99*	0.102
		Educational rights and opportunities		2	3.89*	0.100
		Educational policy		2	3.08	0.081
		Parent and professional training		2	2.25	0.061
		Independence		70		
		Government policy		70		
		Religious involvement		70		
		Love and acceptance		70		
		Equal social rights and opportunities		70		
		Vocational opportunities		70		
		Educational opportunities		70		
		Educational policy		70		
		Parent and professional training		70		

**p < .001, *p < .05,

**Table 7:** MANOVA Examining Differences in Ratings on Likelihood Criterion by Participant Group

Criterion	Predictor	Aggregate Cluster Name	*df*		*F*	Partial ɳ^2^
Likelihood	Part. Type	Independence		2	5.76*	0.122
		Government policy		2	2.38	0.054
		Religious involvement		2	1.42	0.033
		Love and acceptance		2	10.23**	0.198
		Equal social rights and opportunities		2	9.24**	0.182
		Vocational opportunities		2	9.11**	0.180
		Educational opportunities		2	7.57*	0.154
		Educational policy		2	9.88**	0.192
		Parent and professional training		2	3.09	0.69
		Independence		83		
		Government policy		83		
		Religious involvement		83		
		Love and acceptance		83		
		Equal social rights and opportunities		83		
		Vocational opportunities		83		
		Educational opportunities		83		
		Educational policy		83		
		Parent and professional training		83		

**p < .001, *p < .05,

On the importance criterion, there were significant differences in participant group means on the *independence, vocational opportunities*, and *educational rights and opportunities* aggregate clusters. Bonferroni’s post hoc analysis showed that parents rated *independence* higher than healthcare providers, and teachers rated *vocational opportunities* higher than healthcare providers.

On the likelihood criterion, significant differences were evident in the *independence, love and acceptance, equal social rights and opportunities, vocational opportunities, educational rights and opportunities*, and *educational policy and practice* aggregate clusters. Post hoc analysis showed that parents accorded higher ratings on the *independence* aggregate cluster than teachers. Also, parent ratings were significantly higher than both teacher and healthcare provider ratings on the remaining significant aggregate clusters. There were no significant differences between teachers and healthcare providers on the likelihood ratings of any aggregate cluster.

## Discussion

In spite of an increasing number of studies on developmental disabilities, especially in relation to ASD and children with ID, there is generally limited research in Ghana. This is the second study thus far to examine the expectations of care providers within the Ghanaian context. Three key research goals guided this study: (1) we examined service providers’ general expectations for children with ASD or ID; (2) we compared the differences between service providers and parents in the perceptions of the importance of specific expectation domains; and (3) we compared the perceptions of the likelihood of specific expectations being met. We identified different expectations, grouped into nine clusters. These expectations varied between service providers and parents. We found differences among service providers (teachers and healthcare providers) and between service providers and parents on both the importance and the likelihood of expectations being met.

### Ghanaian Service Provider Expectations

Results indicate an almost universal expectation among service providers that children will and should be independent. Independence reflects the ability of the child to communicate their feelings and needs, have the autonomy to make independent decisions, and have those desires respected. Almost all the providers expressed an expectation that children with developmental disabilities should have independence. Consistent with prior work, ratings on the likelihood that this expectation would be met were slightly lower, especially for teachers (e.g., Ivey, 2001). Although most of the statements comprising the cluster may seem apt and intuitive, the statement about “personal faith in the Lord Jesus Christ” might be surprising, as it could be perceived as better if captured under the “*love and acceptance*” or “*involvement of religious institutions*” clusters. For instance, viewing the statement from a more Western lens might lend itself to the “love and acceptance theme” as religious freedom fits within the discourse on diversity and inclusion (e.g., Moon, 2002). However, a critical examination of the Ghanaian context and culture reveals approximately, 71% of Ghanaians identify as Christians, the remainder identify as Muslim (18%), indigenous or animistic (5%), or belonging to other religious groups (6%). Therefore, irrespective of religious affiliation, Ghana is a religious country (Beck & Gundersen, 2016). Consequently, the reference to personal faith and Jesus Christ, in particular, may reflect the Ghanaian religious demographic, the core belief that religion is fundamental to each individual’s identity, and the country’s dominant religious group (Takyi & Addai, 2002).The specificity of the expectation may also have been the personal conviction of the individual focus group participant. However, other participants did not object to its inclusion and participant ratings of this expectation were relatively high, supporting our belief that the expectation was shared among participants. The strong link to personal identity in contrast to familial or societal access or assistance, captured under the “*love and acceptance*” and “*involvement of religious institutions*” clusters, may have prompted its inclusion under the *independence* cluster.

Apart from the overarching independence cluster, two closely related clusters emerged: love and acceptance, and equal social rights and opportunities. Both clusters are defined by a desire for respect and recognition, non-discriminatory behavior towards children with ASD or ID, and the belief that these children must be afforded the same opportunities available to others. While societal attitudes are becoming less antagonistic towards individuals with developmental disorders (both physical and neurodevelopmental) in Ghana, negative attitudes such as stigma and discrimination are not uncommon for children and adults with disabilities in different contexts. For example, discrimination is evident in the fact that resources to support the education of children with disabilities are limited or non-existent even in public schools (Odongo et al., 2021). Employment opportunities are also limited (Agyei-Okyere et al., 2019; Naami, 2015) and the rights of individuals with disabilities, while backed by government legislation and international charters, are not fully implemented (Tuakli-Wosornu & Haig, 2014).

Another set of related constructs is *Educational rights and opportunities*, *Vocational opportunities and protections* and *Government policies*. Government plans and implements educational policies and provides resources for the education of all children and, supports teacher training and the provision of appropriate learning environments. Inclusion appears to be an overarching theme across many clusters; recognition of the rights of the child with developmental disabilities, equal access to social opportunities, educational rights and opportunities, as well as love and acceptance in society. These expectations also highlight gaps in the implementation of disability legislation and represent a call to further action.

### Service Providers’ and Parents’ Perceptions of the Importance of Specific Expectation Domains

Perceptions of importance varied between parents and service providers on two clusters: independence and vocational opportunities. Parents rated independence higher than healthcare providers, and teachers rated vocational opportunities higher than healthcare providers. As compared to service providers, parents were more likely to expect children with developmental disabilities would become independent, care for, and make decisions for themselves. Our findings are consistent with findings from other studies of parental expectations (e.g., Ivey, 2001; Kirby, 2016). It also attests to the value that parents of children with developmental disabilities in diverse cultural contexts place on the attainment of independence by their children.

Unsurprisingly, teachers rated vocational training higher than healthcare providers and parents. The role of teachers is to help children with developmental disabilities acquire vocational skills and therefore, it is natural that their perception of how important this expectation is would be higher. In sum, the findings show that differences exist between service providers and parents in predictable ways depending on the domain.

### Service Providers’ and Parents’ Perceptions of the Likelihood of Children’s Attaining Each of the Identified Expectation Domains

Consistent with findings on importance, parents accorded higher ratings on the independence aggregate cluster than teachers. On the remaining significant aggregate clusters, parent ratings were still higher than both teacher and healthcare providers’ ratings. There were no differences between service providers. Higher ratings for parents may be attributable to optimism. Caring for a child with ASD or ID can be very challenging, leaving parents stressed and in some cases affecting parents’ mental health (Baker et al., 2005; Peer & Hillman, 2014). However, in addition to coping styles and social support, optimism is another important factor that may influence resilience among parents of children with developmental disabilities like ASD and ID (Burke et al., 2020; Peer & Hillman, 2014). Optimism helps reduce parents’ stress levels, increase parental well-being, and may also be linked to better child outcomes in their children (Ekas et al., 2010; Jones & Prinz, 2005; Kurtz-Nelson & McIntyre, 2017).

The noted difference between parents and service providers in this study is instructive when considering inter-group interactions. For children with developmental disabilities especially, parents, teachers, and healthcare providers are critical stakeholders directly influencing their development. As illustrated in Bronfenbrenner’s ecological systems theory, the quality of interactions between these microsystems can affect the child’s development (Bronfenbrenner & Ceci, 1994). And as Gannotti et al. (2004) also found, these differences may also shape expectations for service providers and consequently affect parents’ notions of whether or not these needs have been met. Less optimal interactions stemming from differing perspectives can result in the adoption of conflicting strategies that can negatively impact child outcomes, but collaborative strategies can improve child outcomes.

Consequently, the study has clear implications for interventions and practice in healthcare centres and schools. Interventions and training initiatives in these institutions should consider these differences, outline their potential impact on interactions with parents and service providers, and outline and implement strategies to reduce the potential for conflict while increasing collaboration among these stakeholders. These initiatives may consider targeting stakeholder groups separately to reduce the negative impact of differential power dynamics on the training process and outcomes. Intentional enforcement of objective, practical, policies, and strategies is also critical, as some of these biases may be implicit. This is especially critical in Ghana, as existing disability policies have been criticised for lacking legislative instruments to ensure implementation (Asante & Sasu, 2015).

The discrepancies noted under both criteria also allude to the need for continued efforts to reduce the stigma and discrimination experienced by families of children with ASD and ID in Ghana. The experiences of these children and their families and its impact on their quality of life and general well-being are well-reported in the literature (E.g., Kassah et al., 2012; Oti-Boadi, 2017). Policies that foster greater societal integration and advocacy efforts highlighting the value of these positive outcomes for children with ASD and ID and their families are sorely needed. Moreover, given the importance of religious beliefs in this context, initiatives led by religious institutions might be particularly impactful.

### Strengths, Limitations, and Future Directions

One of the strengths of this study is the use of a mixed-methods strategy to explore service providers’ expectations and compare these with that of parents. However, this study is not without limitations. Of note, we combined expectations of care providers for children with ASD or ID. ASD and ID are different disorders and grouping them likely impacted our ability to observe differences between the two. As explained in the introduction, our decision to combine them was guided by contextual factors and research evidence. For instance, the fact that ASD, especially the severe symptoms requiring substantial support and characterized by levels 2 and 3 in the DSM-V, and ID largely share symptomatology, such as delays in cognitive development, and likely impairments in verbal/communication (5th ed.; DSM–5; American Psychiatric Association, 2013; Pedersen et al., 2017; Thurm et al., 2019). Moreover, although ID is more commonly diagnosed in Ghana (Ghana Statistical Service, 2014), in recent years, there has been a proliferation of Autism centers, particularly in Accra where this study was conducted. This has increased general awareness of ASD in the country, even though specialized knowledge is still lacking. Therefore, it was deemed more likely that service providers would have general impressions of children with ASD and ID instead of specific impressions of children with each condition. Nonetheless, future studies should examine domain and age-specific expectations as these may generate a wider range of expectations based on the diversity within diagnostic categories. Such findings may also have more salient implications for children with these conditions, as the findings from this study may not be generalizable to children with less severe symptoms.

We did not investigate service providers’ existing knowledge of ASD or ID in this study. However, it is plausible that prior knowledge may influence these expectations. Future studies may consider investigating the current level of knowledge among service providers in Ghana and its links with their expectations for children with ASD, ID, and other developmental disabilities.

Another limitation is the relatively small sample size and the focus of the study in one part of the country, the relatively urban south of Ghana. Small sample sizes typically affect the generalizability of research findings. In the future, researchers may consider using larger sample sizes selected from a broader cultural context. It is plausible that lower literacy rates, lower educational attainment and exposure levels, and stronger leanings to indigenous cultural norms outside Ghana’s urbanized contexts (Ansong et al., 2015; Ghana Statistical Service, 2021; Takyi et al., 2021), might engender alternative findings that might necessitate unique strategies for parents and service providers in these areas. Yet, although these findings may not generalize to the rural areas in Ghana, patterns like those identified in our study may emerge in other African cities and LMIC contexts. This highlights the need for additional studies in LMICs.

Further, although we speculate on parental optimism in this study, we did not actively examine this characteristic in the current study. However, given the value of the trait to both parents and children with developmental disabilities like ASD and ID, future investigations, particularly within the Ghanaian context would be helpful. Relatedly, our findings showcasing parental optimism were relative to the service providers’ ratings. However, there is a dearth of literature examining or comparing perspectives across diverse stakeholders. Although the encouraging reports on inclusive education attitudes and practices in countries like Spain and Germany (Monico et al., 2020), might suggest more congruent ratings among diverse stakeholders, it is important to note that discrepancies were identified when Ivey (2001) examined these perspectives in the USA, a developed country with comparable leanings on inclusive practices. Therefore, it is important to examine these perspectives more extensively and in varied contexts to influence contextually appropriate strategies in each country. Lastly, future studies may advance the field by examining links between service providers’ expectations and service delivery to children with developmental disabilities and their families.

## Conclusion

We compared service providers’ and parents’ perceptions of the importance and likelihood of their expectations among children with ASD and ID. We found that expectations may be grouped under nine clusters and for which there were differences between the groups on specific domains. We also found differences in some domains between teachers and healthcare providers. Some findings were unique, whereas others were similar to prior findings. For example, expectations of independence and respect and acceptance seem to be universal among care providers in our study and care providers in other contexts. We also found differences in ratings, which indicate that in some domains, parents’ expectations are different from service providers’ ratings. Although these findings may have implications for training and interventions, disability-specific investigations are needed to inform more targeted intervention strategies.

## Footnotes

1 We acknowledge and respect the preference for identity-first over person-first language. For instance, we know that “autistic children” is a preferred and recommended term within the Autism community, and deaf people is also preferred within the deaf community. In this paper, we generally adopt person-first language because the study broadly includes children with intellectual disabilities, and study authors are not insiders from within any disability community.
